# Supplementary material for: Adenosine Monophosphate-Activated Protein Kinase (AMPK) Phosphorylation Is Required for 20-Hydroxyecdysone Regulates Ecdysis in Apolygus lucorum
Source: Int J Mol Sci. 2023 May 11;24(10):8587. doi: 10.3390/ijms24108587 (PMC10218703; doi:10.3390/ijms24108587)
Supplement: Supplementary file 1 [file ijms-24-08587-s001.zip › ijms-2340587-supplementary.pdf]

Supporting Information: Figure S1. Comparison of predicted N-terminal amino acid sequences of the phosphorylation domains. Table S1: list of the primers used in the study.

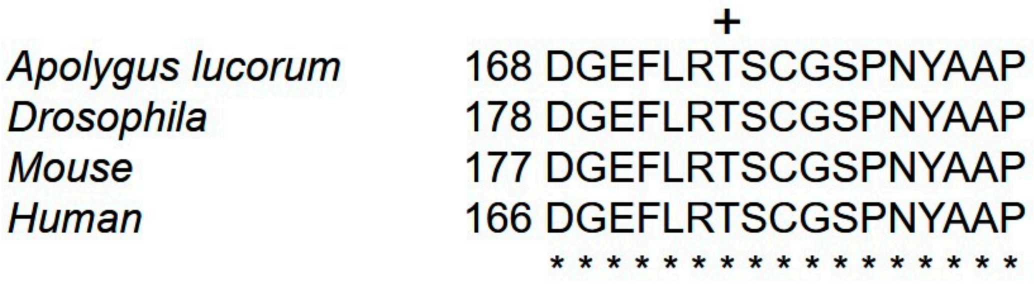

Figure S1 Comparison of predicted N-terminal amino acid sequences of the phosphorylation domains.

Table S1: list of the primers used in the study.

Purpose/primer name	Primer sequences（5′-3′）
cDNA isolation	
AMPK-F	GTKAAAATHGGCGAACATCA
AMPK-F	ACTTCHGGWGCWGCATAGTT
5′- and 3′-cDNA end isolation	
5′- AlAMPK-F	GAATCTCTCGTCGGAT
5′- AlAMPK-R1	ACCGACGACGTCGAGGCT
3′- AlAMPK-R2	CGGCCACTTTGTGTTTGG
3′- AlAMPK-F	CAAGGAGCACGAAGCGAGAAGGTT
3′- AlAMPK-R	ATTGCCACAGGCACATGATCGTTC
qRT-PCR	
AlAMPK-F	CCACGACCAGTTGAGCATAG
AlAMPK-R	GCCTCCTCCTTCTTTGTTGAG
AlEcR-A-F	GGGCAGACGATGACTGGATG
AlEcR-A-R	GTAGCTGTTGCTGGACATAGTTG
AlEcR-B-F	AGGACGGCTGCTGCGATCA
AlEcR-B-R	AGCGAGTCCCAAATCCCAGAG
AlUSP-F	AGCCACCTACACCACCACCTC
AlUSP-R	CTGAGTTGGTGCAGCGGTTGG
AlE75-A-F	TCAGAGCCCGAGAACACC

AlE75-A-R	AGGCGGACGAGTAGAACC
Al-β-Actin-F	ACCTGTACGCCAACACCGT
Al-β-Actin-R	TGGAGAGAGAGGCGAGGAT
RNAi	
dsAlAMPK-F	TAATACGACTCACTATAGGGTACCAAGTCATCAGCACGC
dsAlAMPK-R	TAATACGACTCACTATAGGGATGTGGCAGAGGAGGTTCAC
dsGFP-F	TAATACGACTCACTATAGGGAAGTTCAGCGTGTCCGGCG
dsGFP-R	TAATACGACTCACTATAGGGCACCTTGATGCCGTTCTTCTG

Underlined sequences indicate the T7 RNA polymerase promoter.
